# Peptide-Based Nanoparticles for αvβ3 Integrin-Targeted DNA Delivery to Cancer and Uterine Leiomyoma Cells

**DOI:** 10.3390/molecules27238363

**Published:** 2022-11-30

**Authors:** Anna Egorova, Alexander Selutin, Marianna Maretina, Sergei Selkov, Anton Kiselev

**Affiliations:** 1 Department of Genomic Medicine, D.O. Ott Research Institute of Obstetrics, Gynecology and Reproductology, Mendeleevskaya Line 3, 199034 Saint-Petersburg, Russia; 2 Department of Immunology and Intercellular Interactions, D.O. Ott Research Institute of Obstetrics, Gynecology and Reproductology, Mendeleevskaya Line 3, 199034 Saint-Petersburg, Russia

**Keywords:** non-viral delivery, peptides, iRGD, uterine leiomyoma, pancreatic cancer, integrin, suicide gene therapy, transfection, peptide-based nanoparticles

## Abstract

Uterine leiomyoma is the most common benign tumor of the reproductive system. Current therapeutic options do not simultaneously meet the requirements of long-term efficiency and fertility preservation. Suicide gene delivery can be proposed as a novel approach to uterine leiomyoma therapy. Non-viral vehicles are an attractive approach to DNA delivery for gene therapy of both malignant and benign tumors. Peptide-based vectors are among the most promising candidates for the development of artificial viruses, being able to efficiently cross barriers of DNA transport to cells. Here we described nanoparticles composed of cysteine-crosslinked polymer and histidine-arginine-rich peptide modified with iRGD moiety and characterized them as vehicles for plasmid DNA delivery to pancreatic cancer PANC-1 cells and the uterine leiomyoma cell model. Several variants of nanoparticles were formulated with different targeting ligand content. The physicochemical properties that were studied included DNA binding and protection, interaction with polyanions and reducing agents, size, structure and zeta-potential of the peptide-based nanoparticles. Cytotoxicity, cell uptake and gene transfection efficiency were assessed in PANC-1 cells with GFP and LacZ-encoding plasmids. The specificity of gene transfection via αvβ3 integrin binding was proved in competitive transfection. The therapeutic potential was evaluated in a uterine leiomyoma cell model using the suicide gene therapy approach. The optimal formulation was found to be at the polyplex with the highest iRGD moiety content being able to transfect cells more efficiently than control PEI. Suicide gene therapy using the best formulation resulted in a significant decrease of uterine leiomyoma cells after ganciclovir treatment. It can be concluded that the application of iRGD-modified peptide-based nanoparticles has a high potential for cellular delivery of DNA therapeutics in favor of uterine leiomyoma gene therapy.

## 1. Introduction

Gene therapy holds a great potential to prevent and treat inherited and acquired diseases. The introduction of therapeutic nucleic acids (NA) into the cells in order to eliminate/compensate gene defects or delivery of suicide genes to cause pathological cell death are the basic directions underlying the gene therapy approaches [1]. Gene therapy is studied actively for the treatment of many diseases including inflammatory and infectious diseases, cardiovascular diseases, spinal muscular atrophy and Duchenne muscular dystrophy, cystic fibrosis, acquired immunodeficiency syndrome, etc. [2,3,4,5,6]. However, most gene therapy trials are devoted to both malignant and benign tumor treatment [7]. One of the popular approaches in tumor gene therapy is the delivery of suicide genes inducing apoptosis in target cells. Many suicide gene therapy studies use the herpes simplex virus thymidine kinase gene (HSV-TK) followed by treatment with ganciclovir (GCV) [8]. It should be noted that suicide gene therapy can be successfully used not only for cancer treatment but for reproductive benign tumors as well, including uterine leiomyoma and endometriosis [9,10].

The naked NA is not able to independently enter the cell and reach the cytoplasm, since it is unstable and easily degraded under the influence of extra—and intracellular agents. Thus, viral and non-viral gene delivery systems are applied. Viral vectors have become widespread in gene therapy due to their natural ability to transduce eukaryotic cells. Viral gene therapy utilizes the highly efficient adenoviruses, adeno-associated viruses, lentiviruses or retroviruses [11,12,13]. Currently, viruses are the most efficient gene delivery systems. However, their use is associated with a variety of side effects, in particular with the risk of an immune response and toxicity. Moreover, viral vectors have high cost, difficulty in preparation, and the limited size of the delivered genes [14]. All of these impose a number of restrictions on the use of viral vectors in clinical practice and stimulates the search for cheaper and safer DNA delivery vehicles [15]. 

As an alternative, non-viral carriers based on liposomes, polycations, etc. are being actively developed. They are considered to be safer and cheaper compared to viral vectors with fewer side effects [16]. Advantages of non-viral vectors also include low immunogencity and toxicity, ease of manufacture, and their flexibility in compacting large NA molecules [17]. Today, one of the non-viral delivery methods, which has significant potential, is the use of peptide carriers. Such gene delivery vehicles have been fairly well studied and differ in a number of advantages, such as biodegradability and the ability for easy modification of their structure and amino acid composition [18]. 

Arginine residues are the main components of cell-penetrating peptides, widely spread in viral proteins and play an important role in their membrane translocation [19,20]. The guanidine groups of arginines are involved in hydrogen-bond formation with the phosphate groups on the surface of the cellular membrane that results to the uptake of arginine-rich peptides [21]. On the other hand, the guanidine groups could form hydrogen bonds with the phosphate backbones of NA and strongly compact it, thus providing intracellular delivery [22]. Moreover, the arginine-rich peptides have a biosafety unlike most viruses. After arginine-rich peptide/NA polyplexes enter the cells they could be degraded by endosomal enzymes. One possible way for providing the endosomal escape is the incorporation of histidine residues. Imidazole groups of histidines have pKa values of 6.0, which is significantly lower than that of arginine. That helps to protons scavenging inside the endosomes leading to the proton sponge effect and polyplexes endosomal escape [23]. In addition to NA compacting, cell penetration and endosomal escape, NA has to be efficiently released in intracellular compartments. Cysteine residues in the peptide carrier are used to provide controlled intracellular NA release. Cysteines can be oxidized to form cross-linking disulfide bonds in the polyplexes making them stable in the extracellular environment. However, they are easily disassembled under the reducing intracellular conditions [24]. It should be noted that oxidatively polycondensed cysteine-rich peptides were found to be more effective for gene delivery compared to the matrix polymerization that occurs simultaneously with polyplex formation [25,26,27].

To increase the specificity of gene delivery, the polyplexes are modified with ligands for interaction with cellular receptors. Previously, the iRGD peptide (CRGDK/RGPD/EC) was developed to increase the efficiency of antitumor drug penetration [28]. Like the common RGD ligand, the iRGD peptide has a high binding affinity to αvβ3 and αvβ5 integrins in tumor vasculatures [29]. Compared to RGD, iRGD undergoes proteolytic cleavage with the subsequent formation of a truncated CRGDK/R peptide that loses most of its integrin-binding activity, but acquires a neuropilin-1 (NRP-1) affinity due to its interaction with the conditional C-terminal sequence (CendR) (R/KXXR/K) [30]. NRP-1 is overexpressed on various tumors, so NRP-1-dependent endocytosis results in enhanced tumor penetration providing low toxicity to normal cells [31]. The iRGD ligand has already been recommended as an enhancer for specific tumor-targeted gene delivery [27,32,33].

In our recent study we relied upon the special properties of arginine, histidine, and cysteine residues as well as benefits of oxidatively polycondensed peptides and the high efficiency and specificity of iRGD ligands to both malignant and benign tumor cells [26,33]. We used different molar weight compositions of the RGD1—iRGD ligand-conjugated carrier and arginine-rich cysteine-flanked peptide-based oligomer obtained via oxidative polycondensation to provide both specific tumor targeting and efficient DNA transfection. Physicochemical properties, transport of DNA-complexes into tumor cells, transfection efficiency and specificity, as well cytotoxicity of obtained polyplexes were studied in detail. We used the most specific and efficient DNA-polyplexes in the suicide gene therapy experiment in a cellular model of uterine leiomyoma. The proliferative activity of UL cells as well as the amount of the living cells was assessed after the HSV-TK gene delivery with subsequent GCV treatment.

## 2. Results and Discussion

### 2.1. Design of Carriers

Herein, DNA carriers were developed by the non-covalent mixing of RGD1 peptides modified with cyclic iRGD moiety and oxidatively polymerized R6p peptide-based oligomer [26,27,33]. The unmodified RGD0 peptide was used as a control. Reducible arginine-rich peptides were designed and synthesized by oxidative polycondensation due to their potentially low cytotoxicity and their possibility to trigger DNA release in the reductive cytoplasm of cells and to provide efficient gene delivery [24,26]. The inclusion of histidine to RGD1 and R6p peptides is expected to demonstrate endosomal buffering as well as to be a good spacer in the RGD1 peptide for effective and specific cell binding [23,27]. 

We combined RGD1/RGD0 peptides and R6p oligomers with different mol% content: RGD-R6p-0,25/RGD0-R6p-0,25 carriers were obtained by combining 25 mol% of RGD1/RGD0 and 75 mol% of R6p, RGD-R6p-0,5/ RGD0-R6p-0,5—by combining 50 mol% of RGD1/RGD0 and 50 mol% of R6p, whereas RGD-R6p-0,75/RGD0-R6p-0,75 were obtained from 75 mol% of RGD1/RGD0 and 25 mol% of R6p peptides, accordingly. The studied carriers are demonstrated in Table 1.

### 2.2. Physicochemical Characterization of the Peptide/DNA Complexes

**Figure 1:**
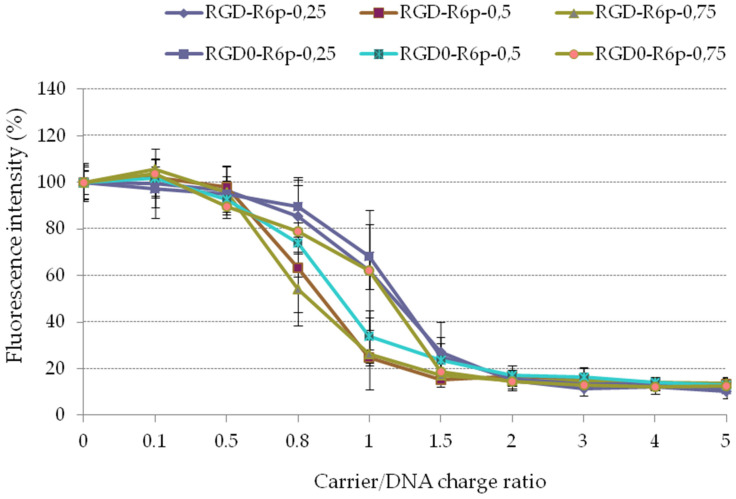
EtBr exclusion assay of DNA complexes with the peptide carriers. Values are the mean ± SD of the mean of triplicates.

The ability of the obtained carriers to condense DNA was tested firstly using an ethidium bromide exclusion assay (Figure 1). The condensation curves for the carriers exhibited the typical transition at a charge ratio of 1.5. The carriers demonstrated efficient condensation capability with low residual fluorescence intensity.

**Figure 2:**
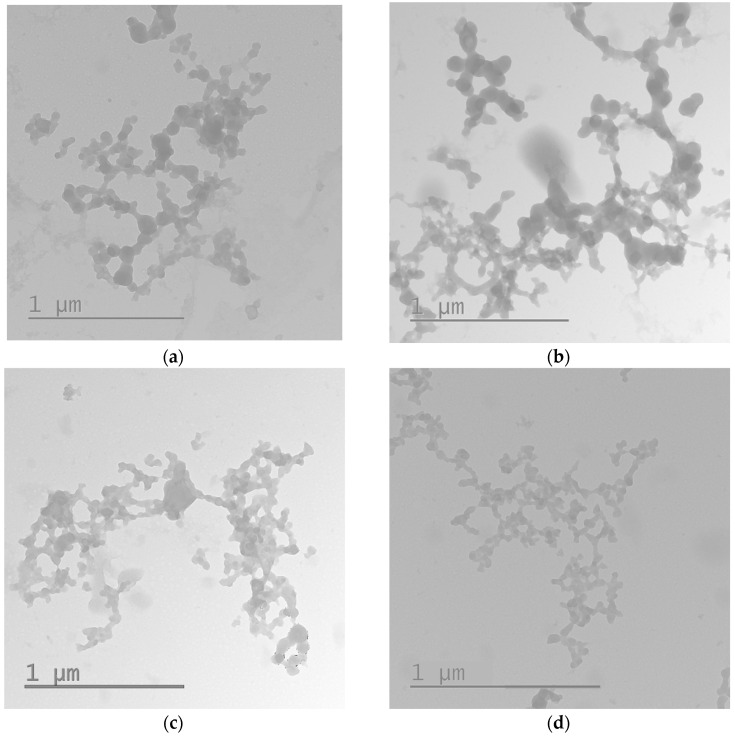
Typical micrographs of the RGD-R6p-0,75/DNA (**a**,**b**) and RGD0-R6p-0,75/DNA (**c**,**d**) polyplexes obtained by transmission electron microscopy: (**a**,**c**) N/P ratio 8/1; (**b**,**d**) N/P ratio 12/1.

The DNA-complexes’ size and their zeta-potential play important roles in determining polyplex stability, gene release behavior, cellular uptake and transfection efficiency. The average diameters of RGD0-R6p-0,25, RGD-R6p-0,25, RGD0-R6p-0,5 andRGD-R6p-0,5 polyplexes were less than 200 nm (Table 2). These polyplex sizes are suitable for penetration via clathrin-dependent endocytosis [34]. The zeta potentials of these complexes were slightly positive (around 13-15 mV), and that allowed them to interact with negatively-charged cell membranes. However, the size of RGD0-R6p-0,75/DNA and RGD-R6p-0,75/DNA polyplexes was larger and varied in the range of 620-970 nm (Table 2). The increase in the polyplex size could be due to the smaller complex aggregation under conditions of excess positive charge with forming larger structures as described [35]. To prove this hypothesis we studied the RGD0-R6p-0,75/DNA and RGD-R6p-0,75/DNA polyplexes formed at charge ratios 8/1 and 12/1 by transmission electronic microscopy. The typical appearance of the studied polyplexes is shown in Figure 2. It can be seen that most of complexes are included to large aggregates but their individual sizes are in the range 70–120 nm (Figure 2). Thus, according to the obtained data, RGD0-R6p-0,75 and RGD-R6p-0,75 carriers can form complexes with DNA that have sizes suitable for cellular endocytosis.

Previous studies on arginine-rich peptide/DNA polyplexes demonstrated that 6000 nm aggregates possess more efficient transfection in comparison with 400 nm polyplexes [36]. Niidome and colleagues supposed that larger complexes might be more efficient in endosomal escape due to stronger membrane perturbation activity [37]. Moreover, the studied RGD0-R6p-0,75/DNA and RGD-R6p-0,75/DNA polyplexes had a neutral zeta-potential that varied from −0.1 to +5.4 mV. This could allow for the avoiding of nonspecific interaction with membranes of other cells and ensure the targeted DNA delivery into tumor ones via iRGD moiety.

**Figure 3:**
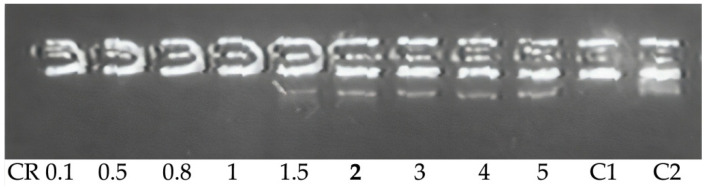
DNase I protection assay of DNA-complexes formed with RGD-R6p-0,75 carriers. N/P ratio in **bold** indicates the beginning of DNA protection. C–, ‘naked’ plasmid DNA treated with DNase I, C+, untreated plasmid DNA.

Since DNA nuclease resistance is important for efficient delivery, we examined the sensitivity of the DNA complex to DNase I (Table 3, Figure 2). The peptide/DNA complexes were exposed to DNase I, and the integrity of the plasmid DNA was analyzed. The data showed that in the polyplexes at 1.5–2 of N/P ratio, DNA was completely protected from DNAse I (Figure 3, lane 6), while the complexes with a smaller amount of peptide were either partially or completely destroyed by nuclease. These results indicate that the carriers are effective for complete protection of DNA against DNase I and, therefore, for protection against similar intracellular nucleases.

**Figure 4:**
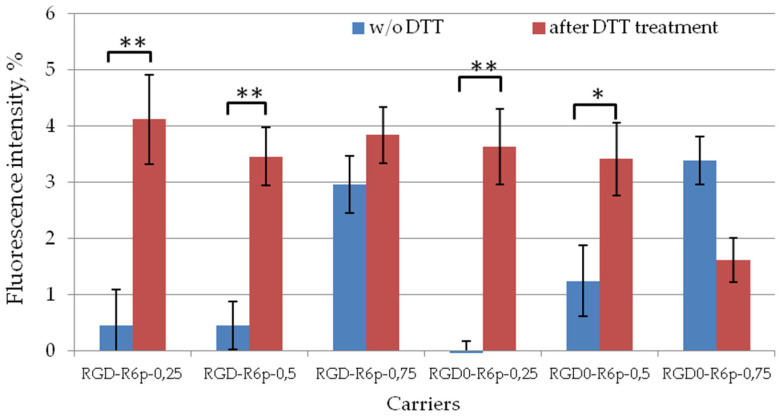
DNA release after DTT treatment of DNA polyplexes. Values are the mean ± SEM of the mean of triplicates. * *p* < 0.05, ** *p* < 0.01 compared to untreated complexes.

The role of cross-linking in polyplex density and stability was evaluated by DTT treatment at 200 nM concentration (Figure 4). DTT is able to reduce disulfide bonds in the same way as cytosol glutathione (GSH) tripeptide [38]. Before DTT treatment, we demonstrated complete DNA condensation by all studied carriers. We observed that DTT treatment resulted in a three-to-eightfold increase in fluorescence intensity in the case of RGD0-R6p-0,25, RGD-R6p-0,25, RGD0-R6p-0,5 and RGD-R6p-0,5 DNA polyplexes. The results obtained testified to the DNA release and the importance of disulfide bonds in the complex formation. However, the level of fluorescence intensity after DTT treatment was significantly lower than that of naked DNA. This testifies in favor of not only disulfide bond reduction but also electrostatic interactions which play a crucial role in cytoplasmic DNA release. On the other hand, in case of RGD0-R6p-0,75/DNA and RGD-R6p-0,5/DNA complexes, DTT treatment did not result in significant DNA release. These polyplexes are 75 mol% composed of non-reducible arginine-rich peptides. We supposed that strong electrostatic bonds between DNA and arginine amino-acids did not allow complex destabilization after disulfide bond reduction in 25 mol% of carrier molecules. 

**Figure 5:**
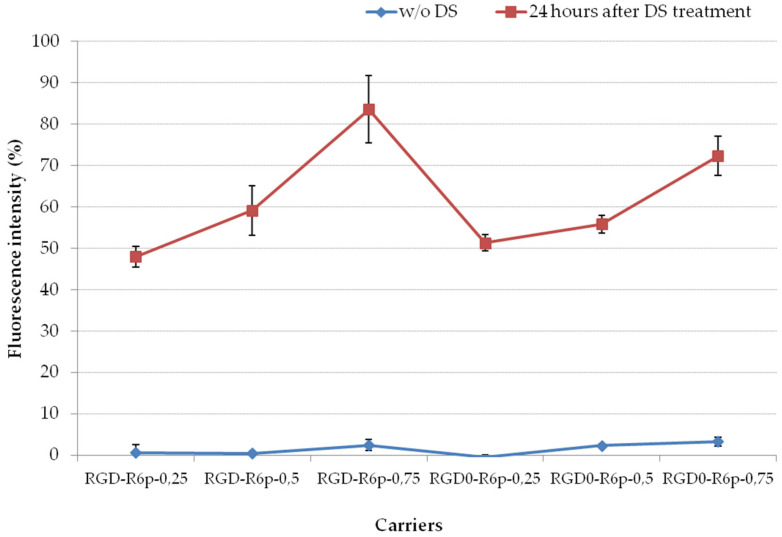
Relaxation of peptide/DNA complexes after 24 h of DS treatment in three-fold charge excess. Values are the mean ± SEM of the mean of triplicates.

The role of electrostatic interactions in cytoplasmic DNA release was proved by complex treatment with negatively charged glycosaminoglycan—dextran sulfate (DS) in three-fold charge excess (Figure 5). Glycosaminoglycans (GAGs) are essential components located on the outer surface of cellular membranes [39]. Polyanionic GAGs interfere with gene delivery by binding to the positively charged complexes. On the other hand, GAGs mediate the binding of the cationic complexes to the cell surface and may act as important receptors for the cellular uptake of polyplexes [40,41]. When inside the cell, the polyplexes need to release DNA and itis an important feature for successful gene transfection which can be realized by interaction with intracellular polyanions. It was shown that 24 h polyplexes incubation with DS resulted in an increase in the fluorescence intensity from 0–2% to 50–80%. The higher DNA release was demonstrated for RGD0-R6p-0,75/DNA and RGD-R6p-0,75/DNA complexes with smaller amounts of cysteine-containing peptides and reached the level of 70–80% compared to naked DNA. 

Thus, the results of DTT and DS treatments indicate that the balance between the charge densities and amount of disulfide cross-linking makes its own adjustments to the predominance of one or another mechanism for the DNA release inside the cytoplasm for efficient gene delivery.

To sum up, the physicochemical characterization of the polyplexes demonstrated that the studied carriers formed stable DNA-complexes, protect DNA from nuclease degradation, and provide DNA release in the cytoplasm of cells. Importantly, no significant variation on DNA-binding, release and protection ability as well as particle size and zeta potential was observed in corresponding non-modified and iRGD-modified polyplexes. The same observations were already noted in our previous studies [27,33]. Since iRGD moiety does not interact with DNA, this gives rise to specific tumor targeting.

### 2.3. Cytotoxicity of Peptide/DNA Polyplexes

**Figure 6:**
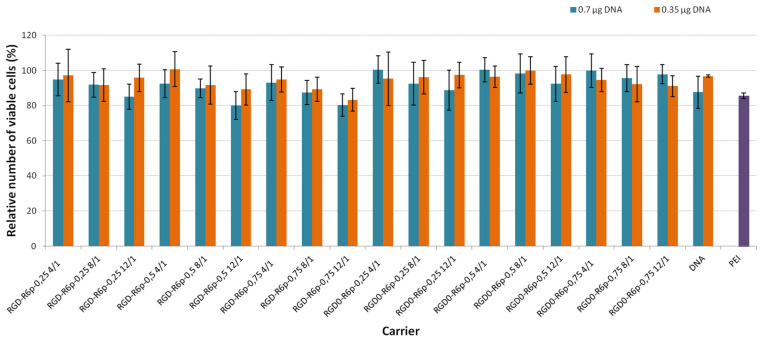
The cytotoxicity after cell transfection with peptide/DNA complexes at 0.7 and 0.35 µg of DNA per well. Values are the mean ± SD of the mean of triplicates.

A successful gene delivery system should be able to deliver genes without negatively affecting normal functions in the cells. In order to use the studied carriers as a DNA delivery agent, the degree of cytotoxicity of formed polyplexes was evaluated. We used DNA in a dose of 0.7 µg and 0.35 µg per well of a 96-well plate which was equivalent to 2 and 1 µg of DNA, accordingly, in a 48-well plate. We planned to use a non-toxic amount of polyplexes in further transfection studies. The cytotoxicity of the DNA-polyplexes was evaluated in PANC-1 cells and compared with cytotoxicity of PEI/DNA complexes (Figure 6). The toxicity of PEI-polyplexes in the range of 20–40% is a well-known fact that has been reported by other researchers [25,42]. The PANC-1 cell line overexpresses αvβ3 integrins with whom iRGD ligand interacts [27,43]. Furthermore, PANC-1 cells were used in the transfection study to prove the efficiency and specificity of iRGD-modified carriers. The results showed that all studied peptide/DNA polyplexes at both DNA dose were equal to or less toxic than PEI polyplexes. The number of living cells after their treatment with peptide/DNA complexes was at least 80%. The results obtained confirmed previous findings of low cytotoxicity of reducible polypeptides [44]. RGD1 and RGD0 peptides were also found to be non-toxic for PANC-1 cells [27]. It is worthy of note that we did not find any differences in cytotoxicity when using both DNA doses. Thus, the studied polyplexes could therefore be regarded as safe for in vitro application, even at higher DNA doses of 2 µg per well.

### 2.4. Cellular Uptake of Peptide/DNA Polyplexes

**Figure 7:**
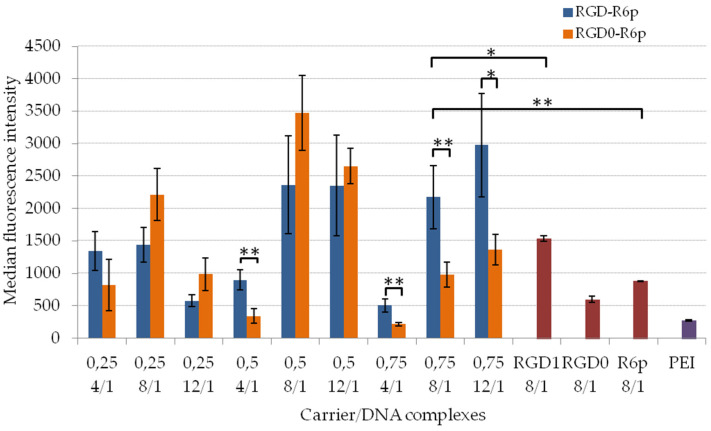
Normalized fluorescence intensity of PANC-1 cells after the uptake of peptide/DNA complexes at 4/1, 8/1 and 12/1 charge ratios labeled with YOYO-1. Values are the mean ± SEM of the mean of triplicates. * *p* < 0.05, ** *p* < 0.01 when compared with R6p/DNA polyplexes.

The cellular uptake of the peptide/DNA polyplexes is crucial to enable effective cell transfection. It might be possible that the increased intracellular uptake of the complexes resulted in enhanced transfection efficiency. In order to examine the effect of iRGD content in the carrier composition on specific cell entry we compared the uptake efficacy of the corresponding ligand-modified and non-modified polyplexes. The cellular uptake of the studied complexes was determined on living αvβ3+ PANC-1 cells by flow cytometry. The normalized fluorescence intensity of YOYO-1-labeled DNA taken up by cells during 2 h is demonstrated in Figure 7. It was shown that the uptake of the studied polyplexes mostly increased with an increase of the N/P ratio. This indicated that a higher surface charge might cause enhanced intracellular uptake. For complexes which were 25 mol% and 50 mol%-modified with iRGD, we mostly did not find any improvements in cell penetration ability if compared with non-modified analogs. It seems to be important to have a balance between ligand content and unspecific transfection efficiency of the carrier to provide targeted gene delivery. A large amount of transfectionally effective R6p peptides in the carrier composition could cause unspecific polyplexes absorption by other cells and tissues. Actually, RGD-R6p-0,75/DNA complexes formed at all studied charge ratios showed significantly higher intracellular uptake efficiency, e.g., 2–2.5 times more than that of appropriate polyplexes without ligands. The obtained results proved that iRGD moiety in this carrier could facilitate the specific attachment of polyplexes by the cells via αvβ3 integrins expressed on the surface of tumor cells [27]. It is worthy of note that RGD-R6p-0,75 and RGD0-R6p-0,75 carriers formed DNA-complexes with near neutral zeta-potential. It can be suggested that these polyplexes can avoid unspecific cell targeting and enter the cell via receptor-mediated endocytosis. Thus, RGD-R6p-0,75/DNA and RGD0-R6p-0,75/DNA polyplexes were chosen for further transfection studies to prove their efficacy and specificity. Other studies also found a higher transfection efficiency of aggregated complexes of larger sizes [36,45].

### 2.5. Transfection Studies

**Figure 8:**
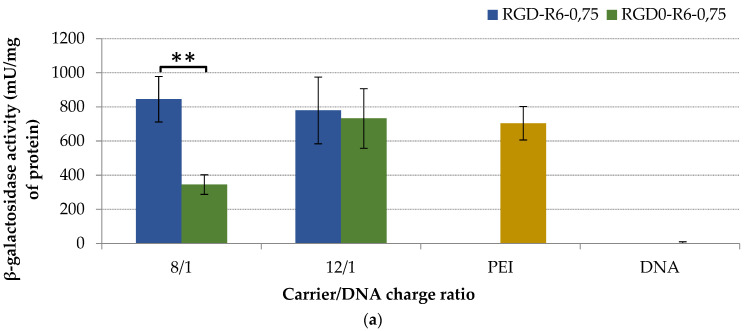

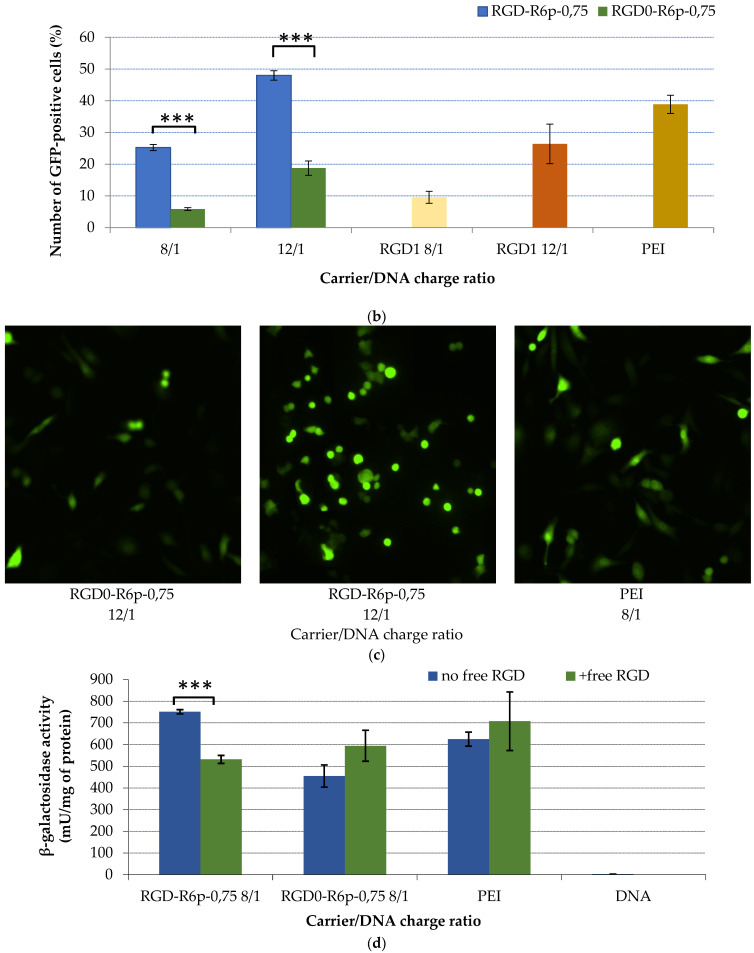

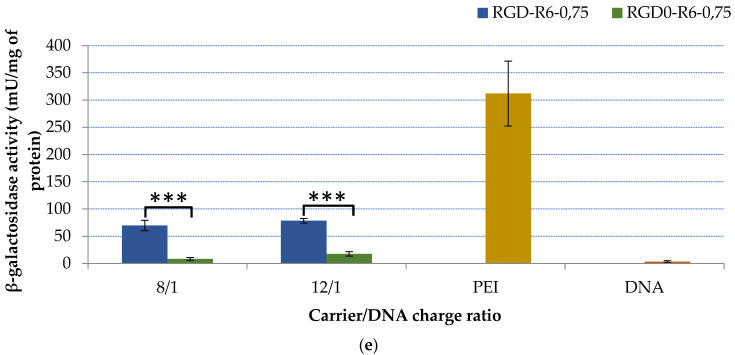
Transfection efficacy studies. The PANC-1 (**a**–**c**) and 293T (**d**) cells were transfected with RGD-R6p-0,75/DNA and RGD0-R6p-0,75/DNA polyplexes at 8/1 and 12/1 charge ratios with pCMV-LacZ plasmid (**a**,**d**,**e**) and pEXPR-IBA5-eGFP plasmid (**b**,**c**). A 10-fold excess of free cRGD molecules were added to the cells for competition transfection studies (**d**). Reporter lacZ gene expression is given as milliunits (mU) per milligram of protein (**a**,**d**,**e**). GFP gene expression is given as a percentage of GFP-positive cells according to flow cytometry (**b**). The typical appearance of GFP-positive cells is shown on fluorescent microscopic images (**c**). Values are the mean ± SEM of the mean of triplicates (** *p* < 0.01, *** *p* < 0.001).

Transfection studies were performed for RGD-R6p-0,75/DNA and RGD0-R6p-0,75/DNA polyplexes. The transfection experiments were conducted to determine the efficacy and specificity of iRGD-modified nucleopeptides to achieve efficient targeted gene delivery with subsequent gene expression in αvβ3-positive PANC-1 cells (Figure 8a–c). Some experiments were carried out in αvβ3-negative HEK293 cells to prove the specificity of ligand-modified polyplexes (Figure 8d). Transfection efficiencies were analyzed by beta-galactosidase expression assay and partly with flow cytometry analysis. As shown in Figure 8a, ligand-modified RGD-R6p-0,75/DNA polyplexes were more effective at DNA delivery than the unmodified RGD0-R6p-0,75/DNA ones at an 8/1 charge ratio. At higher N/P ratios the transfection efficiency of iRGD1-modified and unmodified polyplexes was virtually the same. This could be because of the high efficiency of non-modified DNA complexes to cell transfection. It is known that polyplex-mediated gene transfection is a multistep process which could be highly influenced not only by cellular uptake but also regulated by endosome escape, intracellular DNA release, etc. [25]. However, the transfection efficacy of RGD-R6p-0,75/DNA complexes was comparable or in some cases even higher than that of PEI/DNA polyplexes. The obtained results were additionally confirmed by studies with pEXPR-IBA5-eGFP plasmid via flow cytometry analysis of the transfected cells (Figure 8b). The RGD-R6p-0,75/DNA polyplexes transfected about 25% of cells at N/P of 8/1 and 48% of cells at a 12/1 charge ratio compared to 6% and 18% of GFP(+) PANC-1 cells, respectively, transfected with RGD0-R6p-0,75/DNA. Thus, a flow cytometry analysis showed that iRGD-modified polyplexes were significantly more effective than non-modified ones at both studied N/P ratios. Moreover, RGD-R6p-0,75/DNA complexes containing R6p cross-linking peptide were twice as efficient as non-cross-linked RGD1/DNA polyplexes [33]. Furthermore, the transfection efficacy of RGD-R6p-0,75/DNA polyplexes was comparable or in some cases even higher than that of PEI/DNA (38% of GFP+ cells). The result indirectly demonstrated that the iRGD-modified nucleopeptide complexes were effective and specific for DNA delivery in αvβ3-positive PANC-1 cells. Similar results were obtained in other studies on αvβ3-targeted gene delivery using RGD ligands [27,32,33,46].

Additionally, to demonstrate the specificity of RGD-R6p-0,75/DNA polyplexes for targeted gene delivery we assessed the effect of free cyclic RGD (cRGD) molecules on PANC-1 cell transfection in vitro. We conducted competitive transfection experiments with RGD-R6p-0,75/DNA complexes formed at an 8/1 charge ratio in the presence of a 10-fold excess of cRGD peptide (Figure 8c). The cells’ pre-treatment with cRGD resulted in a 30% decrease in the efficacy of iRGD-modified polyplexes, whereas, the transfection efficiency of RGD0-R6p-0,75/DNA and PEI/DNA complexes did not decrease after the addition of free cRGD. According to the data, we assumed that the iRGD-modified carrier delivered DNA to cells via αvβ3 integrins. 

The nucleopeptide complex transfection might occur not only after targeted gene delivery but also after unspecific electrostatic interactions with the cell membrane. This could be the reason why the transfection efficiency of RGD-R6p-0,75/DNA polyplexes was not decreased to zero in competitive transfections. To prove the specificity of the iRGD-modified carrier we transfected αvβ3-negative HEK293 cells with RGD-R6p-0,75/DNA and RGD0-R6p-0,75/DNA complexes. Importantly, unlike PANC-1 cells herein, the studied polyplexes were significantly less efficient compared to PEI/DNA complexes. Nonetheless, the iRGD-modified carrier was significantly more efficient in DNA delivery than a non-modified one. This might be the result of unspecific polyplex interactions with cell membranes. On the other hand, Oba and colleques previously showed that HEK293 cells expressed a slight amount of αvβ5 integrins which could interact with RGD ligands [43]. The data could help to explain the obtained results. However, the low level of beta-galactosidase gene expression after gene delivery with RGD-R6p-0,75 indicated the inefficiency of the carrier for HEK293 cells.

Taken together, RGD-R6p-0,75/DNA polyplexes seems to be specific and highly efficient for αvβ3-positive cells and can be used in suicide gene therapy studies.

### 2.6. Suicide Effect of RGD-R6p-0,75/pPTK1 Polyplexes with GSV Treatment in Uterine Leiomyoma Cells

Uterine leiomyoma is a common benign tumor of the muscle uterus layer-myometrium. Despite it being a benign tumor, uterine leiomyomas can cause gynecological problems such as uterine bleeding, pelvic pain and infertility, and is the most common reason for uterus removal [47,48]. In addition to hysterectomy, different minimally invasive surgery techniques such as laparoscopy and minimally invasive myomectomy have been developed. However, recurrent new tumor formation remains an important problem in the disease treatment and stimulates the development of new treatment approaches [49,50]. The absence of metastases, the clear localization of myomatous nodes determined by ultrasound, and the availability for various endoscopic techniques make uterine leiomyoma an ideal target for in situ suicide gene therapy.

**Figure 9:**
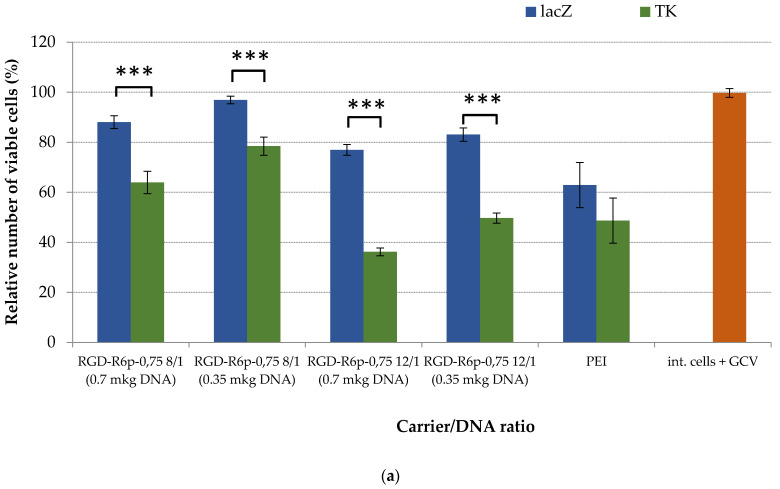

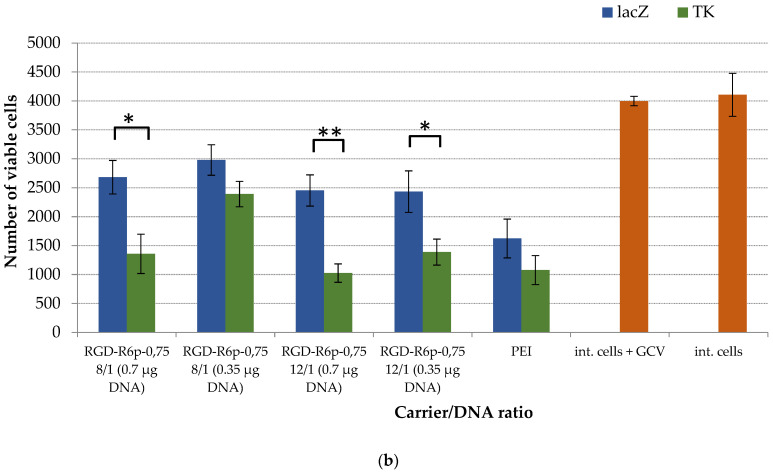
Relative UL cell viability (**a**) and a number of viable UL cells (**b**) after HSV thymidine kinase expression and GCV treatment. Values are the mean ± SD of the mean of triplicates. * *p* < 0.05, ** *p* < 0.01, *** *p* < 0.001 compared to pCMV-LacZ-complexes.

Suicide gene therapy based on HSV-TK transgene and GCV metabolism specificities is widely used to selectively kill tumor cells [51]. To assess the potential use of the peptide DNA delivery vehicles studied here, we transferred HSV-TK genes into primary uterine leiomyoma cells obtained after myomectomy from women with the disease [52]. Our previous studies demonstrated that up to 73% of UL cells are αvβ3-positive [33]. Complexes with pCMV-LacZ served as controls. Following transfection, UL cells were incubated for an additional 4 days with 50 μg/mL of GCV. The relative and absolute number of viable cells was determined using alamarBlue and Trypan Blue assays, respectively (Figure 9a,b).

An alamarBlue assay revealed that after cell transfection with RGD-R6p-0,75-polyplexes and subsequent cell incubation with GCV, the intact cells and pCMV-LacZ-containing cells were insensitive to GCV treatment, whereas the HSV-TK delivery resulted in GCV-related cytotoxicity (Figure 9a). Thus, the suicide effects were demonstrated only for RGD-R6p-0,75/pPTK complexes; we observed a significant 1.2–2.2-fold decrease in UL cells’ proliferative activity depending on the dose of DNA and N/P ratios. The decrease in cell viability caused by control RGD-R6p-0,75/pCMV-LacZ complexes was lower than that of PEI-polyplexes and did not exceed 20%. Moreover, PEI/pPTK1 treatment did not lead to the GCV-related killing of UL cells. A similar tendency was revealed by the trypan blue assay (Figure 9b). The number of viable UL cells transfected with RGD-R6p-0,75/pPTK complexes was significantly decreased to the level of 41–57% compared to pCMV-LacZ polyplexes. PEI/pPTK1 complex delivery did not lead to a decrease in the amount of living cells. Importantly, the suicide effects were more pronounced using RGD-R6p-0,75/pPTK polyplexes at 12/1 of N/P ratio and with 0.7 µg of DNA.

**Figure 10:**
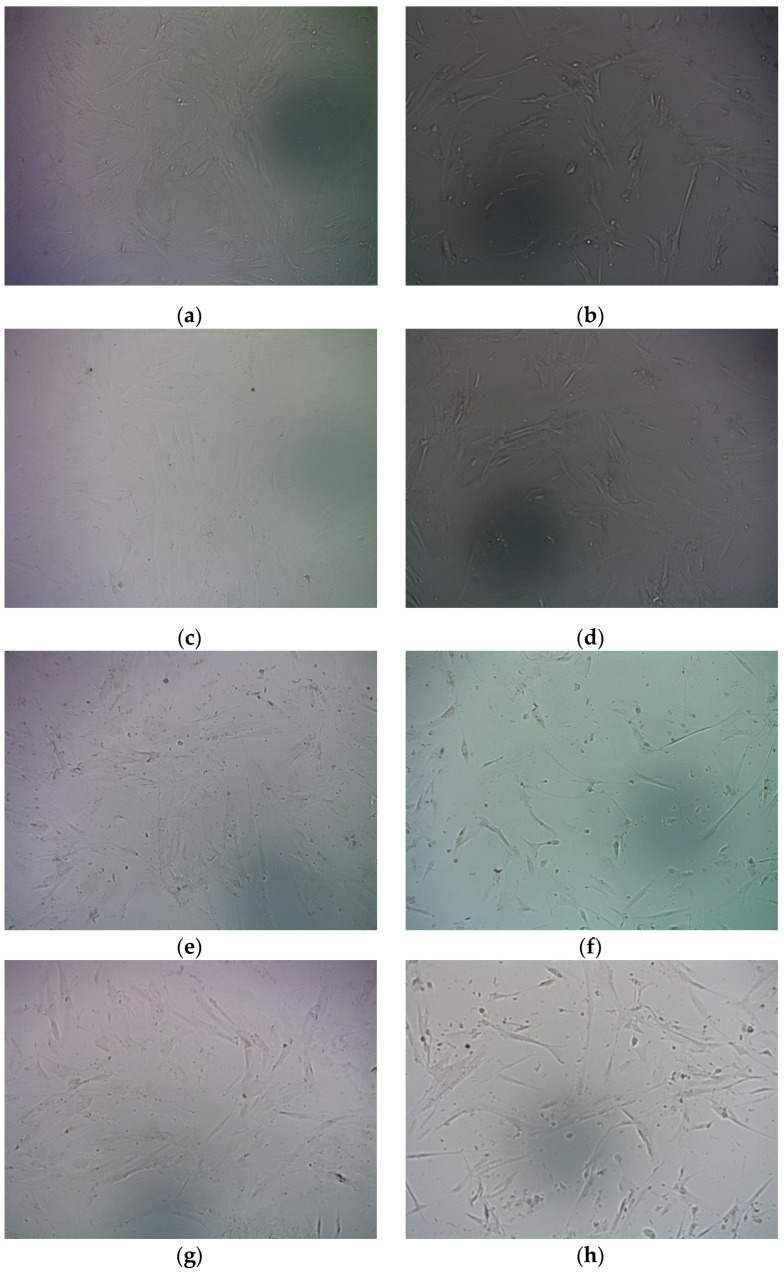

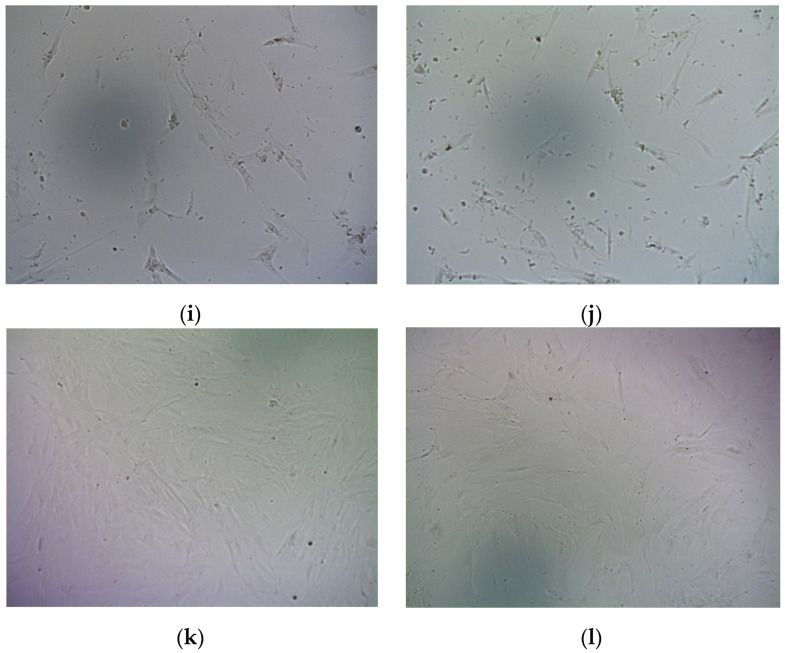
Representative micrographs of UL cells in bright field undertaken 96 h after GCV treatment. (**a**,**b**)—RGD-R6-0,75 8/1 (0.7 µg DNA); (**c**,**d**)—RGD-R6-0,75 8/1 (0.35 µg DNA); (**e**,**f**)—RGD-R6-0,75 12/1 (0.7 µg DNA); (**g**,**h**)—RGD-R6-0,75 12/1 (0.35 µg DNA); (**i**,**j**)—PEI; (**k**)—intact cells; (**l**)—intact cells + ganciclovir. (**a**,**c**,**e**,**g**,**i**)—LacZ encoding plasmid; (**b**,**d**,**f**,**h**,**j**)—TK encoding plasmid.

Accordingly, bright field microscopy visually showed a significant decrease in the amount of cells after their treatment with RGD-R6p-0,75/pPTK polyplexes and GCV treatment compared to controls (Figure 10).

Previously, we developed several gene delivery systems based on matrix polymerized or polycondensed crosslinking peptides modified with iRGD or cycloRGD ligands [33,53,54]. Developed non-viral carriers were used for HSV-TK gene transfer to the cellular model of uterine leiomyoma and suicide gene therapy effects were accessed by different methods including an alamarBlue assay. Based on the current and previous results, these nanoparticles’ efficacy can be compared. According to alamarBlue assay data the most pronounced therapeutic effect—elimination of 57% UL cells was registered for R6p-cRGD/pPTK1 polyplexes; however, the application of RGD-R6p-0,75/pPTK1 and RGD1-R6/pPTK polyplexes was not much less efficient—53% and 46%, respectively [33,53]. In addition, it should be noted that a combination of R6p-cRGD/pPTK1 polyplexes with magnetic nanoparticles decreased the therapeutic effect—only 33% of UL cells were eliminated; however, transfection time was greatly decreased [54]. Thus, it can be concluded that the application of reducible polycondensed R6p carrier has a major impact on the transfection efficacy and HSV1-TK-mediated therapeutic effect. Covalent modification of the reducible polymer with cRGD ligand or non-covalent inclusion of iRGD-modified peptide into composition of the polyplexes also increased the therapeutic effect. It can be suggested that covalent modification of R6p with an iRGD ligand will further increase the gene therapeutic efficacy of pPTK1-bearing polyplexes. The study of such carriers can be a direction for future research of UL suicide gene therapy based on non-viral DNA delivery.

## 3. Materials and Methods

### 3.1. Cell Lines

Human pancreatic (PANC-1) and human kidney (HEK293T) cells were purchased from the Cell Collection of Institute of Cytology RAS (Saint-Petersburg, Russia). The cells were cultured according to the standard method “Fundamental Techniques in Cell Culture” SIGMA-ALDRICH (Sailsbury Wiltshire, SP4, 0JG, UK). Primary UL cells were isolated from myomatous nodes after hysterectomy was carried out in the D.O. Ott Research Institute of Obstetrics, Gynecology and Reproductology (Saint-Petersburg, Russia), as described previously [52]. Briefly, the UL cells were propagated in AmnioMAX C-100 Complete Medium (Thermo Fisher Scientific, Carlsbad, CA, USA) supplemented with 10% FBS (Thermo Fisher Scientific) and 0.3% gentamicin. Only p1 cell culture was used for the transfection experiments.

### 3.2. Peptides Synthesis and Characterization

R6 (*H_2_N*-C-H-(R)_6_-H-C-*COOH*), RGD0 (*H_2_N*-(R)_9_-(H)_4_-*COOH*) and iRGD-modified RGD1 (*H_2_N*-(R)_9_-(H)_4_-C-R-G-D-R-G-P-D-C-*COOH*) peptides were purchased in NPF Verta, LLC (St. Petersburg, Russia). The peptides were synthesized by the solid phase Boc-chemistry method and stored at −20 °C as a dry powder. The purity of the peptides was shown to be 90–95% by high-performance liquid chromatography. The sequences of the peptides and resulting carriers are demonstrated in Table 1. RGD0 was dissolved in ddH_2_O at 2 mg/mL and stored at −20 °C. The oxidative polycondensation reaction of R6 was performed with 2 mg of the peptide at 30 mM concentration containing 30 vol% DMSO and allowed to react at room temperature for 96 h as previously described [26]. The resulting R6p carrier dissolved in water to 2 mg/mL and was stored at −70 °C. RGD1 was cyclized overnight at RT in 0.5 mM Hepes (pH = 7.5) at 0.1 mg/mL, evaporated to 2 mg/mL and stored at −70 °C as described previously [27,55]. The relative amount of free thiol groups in R6p and cyclized RGD1 carriers were estimated by Ellman’s assay as described previously [27].

### 3.3. Reporter Plasmids

The reporter gene expression plasmids pCMV-LacZ encoding β-galactosidase (a gift from professor B. Sholte, Erasmus University Rotterdam, The Netherlands), pEXPR-IBA5-eGFP encoding green fluorescence protein (IBA GmbH, Göttingen, Germany) and pPTK1 with HSV1 herpes virus thymidine kinase gene (a gift from Dr. Orlov from the Institute of Experimental Medicine, St. Petersburg, Russia) were used throughout these studies. Plasmid DNA was grown in Escherichia coli and isolated according to the standard alkaline lysis technique [56].

### 3.4. Nucleopeptide Complexes Preparation and Their Physico-Chemical Characterization

The DNA complexes with peptide carriers were prepared in Hepes-buffered mannitol (HBM) (5% (*w*/*v*) mannitol, 5 mM Hepes, pH 7.5) by mixing the peptide and DNA solutions at various N/P as described previously [26,27]. Complexes were incubated at room temperature for 30 min before using them for physico-chemical and transfection assays. Polyethyleneimine (branched PEI 25 kDa; Sigma-Aldrich) was used as 0.9 mg/mL (pH 7.5) aqueous stock solution, stored at +4 °C. The ratio of PEI to DNA was 8:1.

Condensation of plasmid DNA by peptide carries was determined using an ethidium bromide (EtBr) exclusion assay measured with a Wallac 1420D scanning multilabel counter (PerkinElmer Wallac Oy, Turku, Finland) at 544 nm excitation and 590 nm emission wavelengths and calculated as previously described [57]. The dextran-sulfate (DS) (Sigma–Aldrich, St. Louis, MO, USA) was added to the complexes at three-fold charge excess relative to the carrier for 24 h at RT incubation and analyzed by EtBr exclusion assay. The ability of reducing conditions to destabilize DNA-polyplexes was examined by incubating polyplexes with 200 mM DTT at 37 °C for 1 h followed by a SYBR-Green exclusion assay in a Wallac 1420D scanning multilabel counter at an emission fluorescence of 590 nm (585 nm excitation) [27]. DNA integrity after DNAse I protection assay was analyzed in 1% agarose gel after peptide/DNA polyplexes incubated with 0.5 units of DNase I (Ambion, Austin, TX, USA) for 30 min with subsequent 2 min of DNAse I activation and overnight DNA release in 0.1% trypsin [58].

The hydrodynamic diameters of the complexes were measured by dynamic light scattering and the zeta potential was determined by micro electrophoresis using a zetasizer NANO ZS (Malvern Instruments, Malvern, UK). The peptide/DNA polyplexes were prepared as described above in quantities of 1.2 µg of DNA per sample. The measurements were performed in triplicate at 25 °C in Hepes-buffered mannitol, pH 7.5.

### 3.5. Transmission Electronic Microscopy

Micrographs of the RGD-R6-0,75 and RGD0-R6-0,75 polyplexes formed at 8/1 and 12/1 N/P charge ratios have been obtained by transmission electron microscope Jeol JEM-1400 (JEOL Ltd., Tokyo, Japan). A negative staining of the polyplexes with a 1% aqueous solution of uranyl acetate was used to prepare electronic micrographs.

### 3.6. Cellular Uptake of Peptide/DNA Complexes

PANC-1 cells were seeded at a density of 6 × 10^4^ cells/well in 48-well plates for 24 h. Peptides/DNA complexes were prepared with YOYO-1 iodide (one molecule of the dye per 50 base pairs of polynucleotide) labeled pCMV-LacZ at 8/1 and 12/1 charge ratios. At the day of experiment, the cells were rinsed with serum-free medium, and serum-free medium was added to each well. The cells were treated with polyplex solution containing 2 µg of plasmid DNA for 2 h at 37 °C. The cells were then washed twice with cold 1 × PBS (pH 7.2), once with 1M NaCl (in 1 × PBS) and twice again with 1 × PBS. Cells were collected by trypsinization (trypsin-EDTA solution—Biolot LLC, Saint-Petersburg, Russia) and resuspended in 1 × PBS. The cells were incubated with 5 µL of propidium iodide staining solution (50 µg/mL in 1 × PBS) for 15 min in the dark. The degree of cellular uptake was immediately measured by BD FACS-Canto II (BD, Biosciences Immunocytometry Systems, San Jose, CA, USA). The results were presented as RFU/cell in 10,000 cells. 

### 3.7. Gene Transfer 

PANC-1 and HEK293T cells were seeded on 48-well plates at 5.0 × 10^4^ cells/well and transfected the next day at 80% confluency. Prior to transfection, the media were removed and cells were rinsed twice with transfection media (DMEM without FBS). Cells were filled in with 0.5 mL of transfection media containing the peptides/DNA complexes at a concentration of 2 mg DNA/well for 4 h at 37 °C. The transfection media was then removed, and 0.5 mL of fresh DMEM, containing 10% FBS, was added. The cells were incubated for an additional 48 h.

The β-galactosidase activity in cell extracts was measured using methyl-umbelliferyl-β-D-galactopyranoside (MUG) solution with normalizing by the total protein concentration measured with Bradford reagent (Helicon, Moscow, Russia) as described previously [26]. 

A competition study was performed in order to measure iRGD ligand-mediated cellular uptake. Before 15 min of complex treatment, a 10-fold excess of free cyclo(RGDfK) peptides (NPF Verta, Saint-Petersburg, Russia) was added to the cells, followed by the procedures described above. 

The percentage of GFP-positive cells was determined by flow cytometry using a BD FACS-Canto II after cell transfection with pEXPR-IBA5-eGFP plasmid. Micrographs of GFP-positive cells were taken using a Leica DM 2500 microscope (Wetzlar, Germany) with a Leica DFC345 FX camera at ×200 magnification.

### 3.8. Cytotoxicity Assay 

The cytotoxicity of the polyplexes was measured by an alamarBlue assay (BioSources International, San Diego, CA, USA). PANC-1 cells were seeded in a 96-well plate at 2 × 10^4^ cells per well in 100 µL DMEM medium containing 10% FBS. Cells achieved 80% of confluence after 24 h were exposed to polyplexes prepared at the rate of 0.7 or 0.35 µg of DNA per well (equivalent to 2 and 1 µg of DNA in 48-well plate, respectively). The experiment was carried out as described previously [58]. The fluorescence intensity was measured in a Wallac 1420D scanning multilabel counter in emission fluorescence at 590 nm (544 nm excitation) and recorded as a percentage relative to the value of untreated control cells. 

### 3.9. Suicide Gene Therapy

The primary UL cells were seeded in 96-well plates at 1.5 × 10^4^/well in complete media at 37 °C. The next day the transfection was carried out in a serum-free medium with 0.7 µg and 0.35 µg of pPTK1 or LacZ plasmids per well. After 2 h of cells incubation the transfection media was replaced with fresh DMEM containing 10% FBS. Twenty-four hours after this, the media was replaced again with complete media containing GCV (50 μg/mL). The plates were incubated for another 96 h at 37 °C as described previously [33]. 

After four days of incubation the medium was replaced for the fresh one with added 10% AlamarBlue solution and the cells were allowed to grow for 2 h. Fluorescence intensity was measured in a Wallac 1420D scanning multilabel counter in emission fluorescence at 590 nm (544 nm excitation) and the cell proliferation activity was estimated as a percentage relative to the value of intact cells. Cell photos were taken at 100× magnification using an AxioObserver Z1 microscope (Carl Zeiss, Oberkochen, Germany) equipped with the AxioVision program. The total number of living cells after 96 h of incubation was estimated by the trypan blue exclusion method, as described previously [33].

### 3.10. Statistical Analysis

Statistically significant differences were obtained with the Mann–Whitney U-test and Student’s *t*-test using Instat 3.0 (GraphPad Software Inc., San Diego, CA, USA). *p* < 0.05 was considered statistically significant.

## 4. Conclusions

In the presented study we demonstrated the efficient and specific non-viral gene delivery to both malignant and benign tumor cells by means of iRGD ligand-modified nanoparticles. We have shown that the inclusion of polycondensed arginine-histidine-rich oligomers in the composition of DNA-peptide nanoparticles greatly improves their transfection properties; however, it was found that the specificity of DNA transfer to cells is strongly dependent on the ligand content. The cellular model of uterine leiomyoma was used to demonstrate the therapeutic potential of the best developed formulation. The proliferative activity of UL cells and the amount of the viable uterine leiomyoma cells were significantly decreased after HSV-TK gene delivery with subsequent ganciclovir treatment.

The developed suicide gene delivery system based on iRGD ligand-modified peptide nanoparticles can be suggested as a useful tool for the development of cancer and uterine leiomyoma gene therapy.

## Figures and Tables

**Table 1:** Design and composition of the carriers.

Name	Composition (mol%)
RGD0-R6p-0,25	RRRRRRRRRHHHH (25 mol%) + (-CHRRRRRRHC-)n (75 mol%)
RGD-R6p-0,25	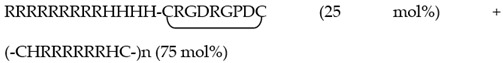
RGD0-R6p-0,5	RRRRRRRRRHHHH (50 mol%) + (-CHRRRRRRHC-)n (50 mol%)
RGD-R6p-0,5	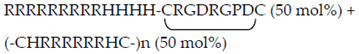
RGD0-R6p-0,75	RRRRRRRRRHHHH (75 mol%) + (-CHRRRRRRHC-)n (25 mol%)
RGD-R6p-0,75	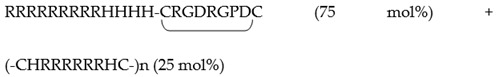

**Table 2:** Size and zeta-potential of carrier/DNA complexes at N/P ratio 8/1. The data are shown as the mean ± S.D.

Carrier	Size ± SD	Z-Potential ± SD
RGD-R6p-0,25	102.7 ± 2.81	14.9 ± 2.57
RGD-R6p-0,5	165.6 ± 0.51	13.4 ± 0.61
RGD-R6p-0,75	627.2 ± 18.36	−0.1 ± 0.26
RGD0-R6p-0,25	94.9 ± 2.50	13.9 ± 1.04
RGD0-R6p-0,5	104.0 ± 4.61	13.6 ± 2.50
RGD0-R6p-0,75	968.7 ± 45.64	5.4 ± 0.25

**Table 3:** DNase I protection ability of peptide/DNA polyplexes.

Carrier	Carrier/DNA Charge Ratio
RGD-R6p-0,25	2/1
RGD-R6p-0,5	2/1
RGD-R6p-0,75	2/1
RGD0-R6p-0,25	2/1
RGD0-R6p-0,5	1.5/1
RGD0-R6p-0,75	2/1

## Data Availability

The data presented in this study are available on request from the corresponding author. The data are not publicly available due to restrictions of the subjects’ agreement.

## Sample Availability

Samples of the compounds are available from the authors.
